# Physiological Candles

**Published:** 1889-11-30

**Authors:** 


### The Doctor's Pulpit.

### PHYSIOLOGICAL CANDLES.

### "Then" and "Now."

"Then" life was young, and fresh, and confident; and the liver had not put in its plea for the premiership of the body physiological. That organ, which is the master of those spirits that count melancholy and depression in their train, has since asserted its claims without ceasing, and, like all persistent creatures which have necessary work to do, and will only do that work on their own terms, it has gained its point, and is now in many human organisms the first minister of the crown. That is one of the fundamental differences between "then" and "now." The body, however, even in the "then" times of youth, had its prime minister. That active potentiality was of a different temper from the liver. We may call it the principle or the energy of growth or development. That principle or energy was by no means a melancholy spirit. On the contrary, life, movement, effervescence, bounding and resistless animation were its characteristics. Have we done well to depose it from the premiership and to appoint in its place the miserable and melancholy liver? Or has the liver itself usurped the leadership by taking advantage of our physiological ignorance and stupidity, and our general unfitness for discovering and steadily following the most rational ways of life.

If physiology can furnish us with a candle which will light us to any principle of development or growth of a permanent kind, and if we have the good sense to adopt that principle as the *aut vitam aut culpam* premier of the body and the mind, and to remain loyal to it throughout the whole of life, we shall be inclined to look upon physiological science with new and more favourable eyes. But that is exactly what a rational physiology does for us. It shows us that there is a principle of growth or development which is destined by Nature to preside over and animate the whole of life, and that that principle is the only legitimate ruler of the physical life, and that the melancholy and miserable liver is a base usurper, without any claim to leadership, except what has been gradually filched from man's ignorance and indifference.

The principle of growth and development should control and inspire the human organism throughout the whole of life. If it had always done so the life of man would not now be of its present leaden complexion, but would be full of colour, of brightness, and of pleasure. Nobody will contend that childhood and youth are not, on the whole, periods of energetic delight. The average boy runs over with joyous spirits in all directions, and the healthy girl has very much in common with the average boy. Why is the boy happy and the man miserable? Because the energetic principle of growth and development carries the boy triumphantly through and over all sorts of troubles as if he were mounted on the back of a royal eagle; whilst the dull and morose liver holds dreadful and despotic sway over the days and nights of the man of middle age.

But it is argued that the principle of growth and development has expended itself in raising the boy to manhood; and that, therefore, there can be none of the exuberant energy of boyhood in the grown man. It is exactly here where our physiological candle shows its value. Using it, and peering about diligently by means of its light, we find that the principle of growth and development has not expended itself in raising the boy to manhood and the girl to adult age. Far from it; it has only localised its seat of government a little more definitely. In early life the principle of growth and development spends its energies chiefly on the muscles, bones, and similar parts; but when bodily growth is completed, then the energy of development is gradually transferred more to the brain, to the intellect, and the moral faculty. Is this not scientifically and absolutely true? Exactly, therefore, as the energy of physical development carries boys and girls joyously and triumphantly through the cares, anxieties, and difficulties of early life, so also, but in a much more exalted and splendid degree, should the principle of development, which has transferred its chief energies to the brain, carry the grown man through all his labours and difficulties, so that he shall be no more conscious of the presence of liver, heart, and lungs than is the healthy boy.

"But," says the objector, "as a matter of fact the brain does not do this." No, because modern scientific men, with all their pride of science, have not yet learnt even the alphabet of making the highest and best use of the human brain and body. They are at the stage of the old woman who killed the goose that laid the golden eggs. They kill the brain before it is mature; they pluck the fruit before it is half ripe, and then they are surprised at its sourness. They turn the brain into a "cramming" machine instead of a "thinking" organ; and then they wring their hands because the liver puts it on one side and asserts its right of government and authority. Take, for example, so brilliant a man as Carlyle. It is impossible to predict, even yet, whether his fame as a man of letters or his notoriety as a cantankerous and bilious dyspeptic will last longest in the world. With Shakespeare it was quite otherwise. Happily for his fame, and the world's enduring advantage, his early education was neglected. Of another man equally great and famous in English literature, John Bunyan, it may be said with truth that it is doubtful whether he had any early education at all. But what a rare and noble genius was his!

The healthy, active, vigorous, and joyous brain is the developed successor of the youthful principle of growth, and should, in middle and old age, naturally take its place. It would do so if schoolmasters, and parents, and doctors, and men of science were half as wise and clever as they think they are. But parents, schoolmasters, doctors, and men of science set before youths and young men certain objects to be achieved. They make the objects everything and the achiever nothing. The objects are justified on the ground that they are for the benefit of the achiever. But how can they benefit him if they kill him; or if, stopping short at his death, they disable his brain, and place him under the more or less complete sway of the miserable and mindless liver? A certain number of men, whom educationists and ambitious parents, and medical watchdogs who cannot bark, have done their best to kill and destroy, have the sense to see that modern haste to be rich or famous, and modern competitions of all sorts, have the seeds of madness and death in them. These wiser few throw off their allegiance to modern struggles and hurries; and, possessing their souls in proud patience, work steadily, cultivate health, enjoy life, and wait for fame and fortune to come in a legitimate way and at a legitimate time. Such men are not old, broken-down, liver-ruled, and gout-destroyed creatures at fifty. They are still young; and look forward to being more hale and hearty at seventy than the man who groans under the tyranny of his liver is at forty-five.

A few people will smile when they are calmly told that this is a question of imperial importance. It is, however, a question to be classed with the Education Act, and other public movements of like significance. The weakening of the brain in youth and early manhood, and the consequent unhealthy inequality which is brought about between it and the liver, has many and serious results, which no one but a practical physiologist will believe in. It destroys life's natural happiness completely; and the destruction of happiness means an impairment of efficiency which is incalculable. It handicaps future generations heavily, so heavily that the uninstructed man can form no conception of it. But even a fool must see that for a father to pass on to his son a ruined and enfeebled brain, a slow and obstinate liver, a dyspeptic and miserable stomach, is to give him an inheritance for which no wealth of lands or fame can be any adequate compensation. The subject demands thought, and if we could think clearly enough we should see that it demands action, too. The candles of physiology never distort images or reveal ghostly phantoms instead of solid realities. Happy is the man who, having one of these candles, strives to walk in its light.

